# Left ventricular outflow tract velocity time integral outperforms ejection fraction and Doppler-derived cardiac output for predicting outcomes in a select advanced heart failure cohort

**DOI:** 10.1186/s12947-017-0109-4

**Published:** 2017-07-03

**Authors:** Christina Tan, David Rubenson, Ajay Srivastava, Rajeev Mohan, Michael R. Smith, Kristen Billick, Samuel Bardarian, J. Thomas Heywood

**Affiliations:** Fellow, Scripps Clinic Cardiology, 10666 N. Torrey Pines Road, La Jolla, CA 92037 USA

**Keywords:** Congestive heart failure, Velocity time integral, Time velocity integral, Echocardiography

## Abstract

**Background:**

Left ventricular outflow tract velocity time integral (LVOT VTI) is a measure of cardiac systolic function and cardiac output. Heart failure patients with low cardiac output are known to have poor cardiovascular outcomes. Thus, extremely low LVOT VTI may predict heart failure patients at highest risk for mortality.

**Methods:**

Patients with heart failure and extremely low LVOT VTI were identified from a single-center database. Baseline characteristics and heart failure related clinical outcomes (death, LVAD) were obtained at 12 months. Correlation between clinical endpoints and the following variables were analyzed: ejection fraction (EF), pulmonary artery systolic pressure (PASP), NYHA class, renal function, Doppler cardiac output (CO), and LVOT VTI.

**Results:**

Study cohort consisted of 100 patients. At the 12-month follow up period, 30 events (28 deaths, 2 LVADs) were identified. Occurrence of death and LVAD implantation was statistically associated with a lower LVOT VTI (*p* = 0.039) but not EF (*p* = 0.169) or CO (*p* = 0.217). In multivariate analysis, LVOT VTI (*p* = 0.003) remained statistically significant, other significant variables were age (*p* = 0.033) and PASP (*p* = 0.022). Survival analysis by LVOT VTI tertile demonstrated an unadjusted hazard ratio of 4.755 (CI 1.576-14.348, *p* = 0.006) for combined LVAD and mortality at one year.

**Conclusions:**

Extremely low LVOT VTI strongly predicts adverse outcomes and identifies patients who may benefit most from advanced heart failure therapies.

## Background

Congestive heart failure is persistently growing health care dilemma in the United States, resulting in more than 50,000 deaths annually and more than 2 million yearly hospitalizations [1, 2]. The clinical assessment of patients with heart failure has been shown to be a valuable tool in guiding therapy and predicting prognosis, with the presence or absence of congestion and hypoperfusion dividing heart failure patients into one of four hemodynamic profiles [3]. Patients with both congestion and low cardiac output have been shown to have more than twice the mortality and cardiac transplantation rates of those without hypoperfusion and congestion [3]. Thus, accurate and timely identification of high risk profiles is crucial. Due to the burden of disease, as more patients with heart failure are cared for by clinicians without expertise in advanced heart failure, an easily utilized screening tool for identifying such patients would be helpful to facilitate timely assessment for advanced therapies such as left ventricular assist device and cardiac transplantation [4].

Echocardiography has emerged as an invaluable tool in the diagnosis and management of congestive heart failure (CHF) and is routinely performed in patients with CHF [5]. Measurements of left and right ventricular dysfunction, presence of valvular disease, Doppler derived cardiac output, and estimates of intracardiac pressures are frequently obtained metrics [6]. Multiple studies have demonstrated a close correlation between cardiac output calculated by Doppler echocardiography and invasive thermodilution and Fick methods [7, 8]. Doppler derived cardiac output is typically obtained by measuring flow across the left ventricular outflow tract (LVOT) which is determined by the velocity time integral of the Doppler signal directed across the LVOT (LVOT velocity time integral or LVOT VTI), multiplied by the cross sectional area of the LVOT and heart rate. LVOT VTI has been shown to be a reproducible measurement even in the context of severe chronic heart failure [8, 9] and is superior to flow measured at other locations, including the right ventricle, pulmonary artery, mitral valve, and aortic arch [10] due to multiple factors: ability to obtain insonation parallel to blood flow and a relatively flat profile of blood velocity distribution [11].

Because estimation of the area of LVOT represents the major source of error in deriving cardiac output due to the elliptoid shape of the LVOT and squaring of the measured radius (*πr* ^2^) [12, 13], using LVOT VTI alone rather than Doppler derived cardiac output has been suggested as a reliable surrogate for cardiac output in the absence of left ventricular outflow tract abnormalities [9]. Prior studies have evaluated LVOT VTI in acute myocardial infarction, demonstrating 100% one-month survival for subjects with LVOT VTI greater than 100% predicted for age and greater than 80% survival at 5 years. In contrast, mortality rate at one month and five years were 18% and 43% respectively when LVOT VTI was less than 65% predicted [14]. More recently, a study of 990 patients with stable coronary artery disease demonstrated increased rates of heart failure hospitalization for subjects within the lowest VTI quartile [15]. Our study was designed to test the following concept: for those within the lowest VTI quartile, is extremely low VTI a marker for patients at highest risk of death and who will go on to need advanced therapies such as transplant or LVAD? We hypothesized that because extremely low LVOT VTI is an accurate marker of the low output state, LVOT VTI may be used to discriminate between early stage heart failure versus advanced heart failure with low cardiac output, thus providing clinicians with a readily obtainable noninvasive tool to identify patients who may benefit most from advanced heart failure interventions such as LVAD and heart transplantation.

## Methods

LVOT VTI is used to estimate stroke volume since it reflects the column of blood which moves through the LV outflow tract during each systole, per the following equation [16]:

$$ \mathrm{Stroke}\ \mathrm{Volume}=\mathrm{LVOT}\ \mathrm{VTI}\times \mathrm{Cross}\ \mathrm{Sectional}\ \mathrm{Area}\ \mathrm{of}\ \mathrm{the}\ \mathrm{Left}\ \mathrm{Ventricular}\ \mathrm{Outflow}\ \mathrm{Tract}. $$

**Fig. 1:**
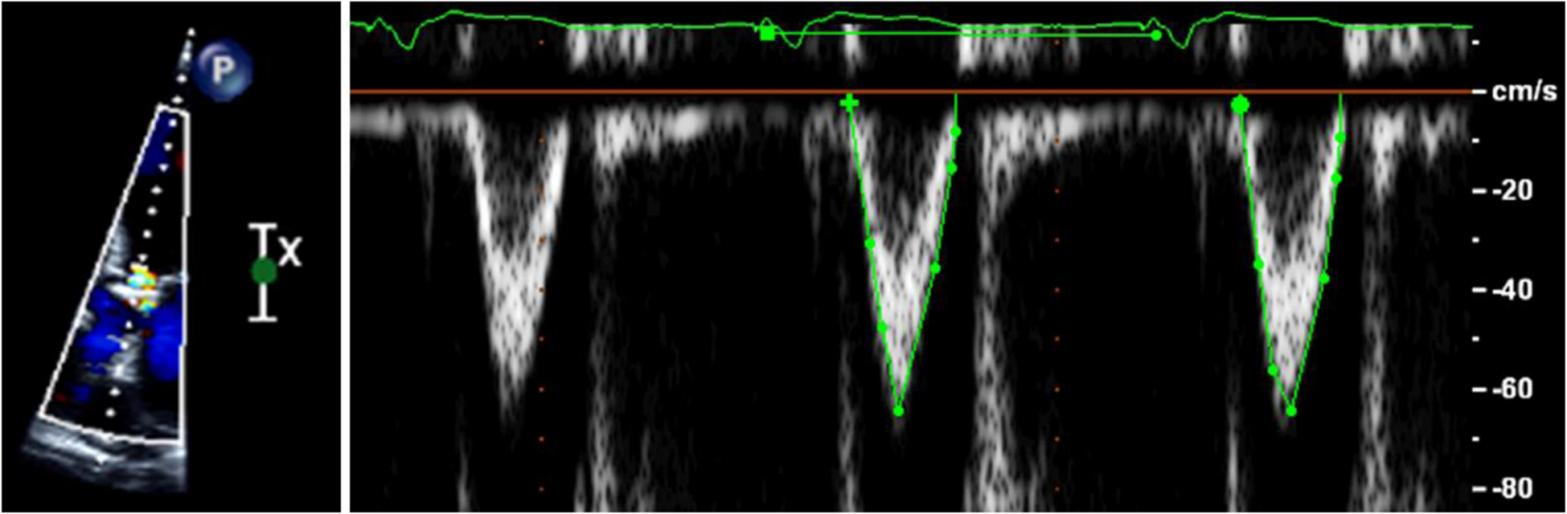
Left ventricular outflow tract stroke distance measured by pulsed wave Doppler echocardiography. Legend: Doppler signal from the apical view is directed parallel to flow through the left ventricular outflow tract and velocity time integral measured by hand planimetry

LVOT VTI is calculated by placing the pulsed Doppler sample volume in the outflow tract below the aortic valve and recording the velocity (cm/s). When the velocity signal is integrated with respect to time, the distance blood moves with each systole is calculated in cm/systole (Fig. 1). Assuming laminar flow through the LVOT, this has been shown to correlate well with cardiac output, which is equivalent to stroke volume x heart rate [16].

The study protocol was approved by the Institutional Review Board of Scripps Health, La Jolla, CA. Subject selection: The transthoracic echocardiogram (TTE) database at our institution was queried for studies performed between June 2009 and May 2011 for patients with heart failure. As over 20,000 echocardiograms per year are performed at our institution, in order to identify subjects with extremely low LVOT VTI, a cutoff LVOT VTI of no more than 10 cm was applied in order to limit the number of included studies.

Exclusion criteria included any one of the following: alternative causes for shock (including hypovolemia and sepsis), acute myocardial infarction, significant valvular disease affecting accurate estimation of forward cardiac output by LVOT VTI (i.e, severe aortic regurgitation) [5], significant tachycardia (defined as >120 beats per minute), and pulmonary arterial hypertension. Subjects with multiple TTEs were reviewed at time of earliest study and subsequent studies were excluded. Valvular disease was characterized according to guidelines from the American College of Cardiology [17]. Diagnosis of heart failure was established by review of the electronic medical record (EMR): clinical documentation of heart failure in an admission history and physical, discharge summary, or cardiologist’s note and/or referral to a dedicated heart failure clinic.

### Echocardiographic measurements

LVOT VTI was measured from an anteriorly angled apical four-chamber view using pulsed wave Doppler with the interrogation beam directed across the LVOT. The pulsed wave Doppler sample volume was placed in the left ventricular outflow tract just proximal to the aortic valve and thin spectral envelopes were obtained using very low gain. In patients with atrial fibrillation, LVOT VTI was averaged over three to five consecutive beats. In cases of aortic stenosis, the pulsed Doppler sample volume was placed far enough from the stenotic valve to avoid falsely elevated readings. Inter- and intra- observer variability testing for LVOT VTI was performed and validated (intra-class correlation coefficient 0.989-0.997). Ejection fraction (EF) was calculated using bi-plane method of disks from apical two and four chamber views. Pulmonary artery systolic pressure was calculated from Doppler derived tricuspid regurgitation velocity using the simplified Bernoulli equation (△*P* = 4*v* ^2^). Doppler derived cardiac output was calculated using the previously described equation for Doppler derived stroke volume, followed by multiplication by heart rate (i.e. CO = SV x HR).

Data collection: Baseline demographic information and laboratory data at time of TTE were obtained from the EMR via retrospective chart review.

### Statistical analysis:

The primary endpoint was defined as death, transplant or LVAD placement within one year of TTE and identified by review of our institution’s EMR and the Social Security Death Index. The study population was analyzed in cohort design based on subjects meeting the primary endpoint versus event-free subjects. Differences in categorical and continuous variables were analyzed by chi-squared and independent t-test analyses respectively.

### Survival analysis

The prognostic ability of various factors including LVOT VTI to predict the primary endpoint over 12 months following TTE was tested by Cox regression analysis. Univariate Cox regression was performed using a set of predefined variables: age, gender, presence of renal dysfunction, hemoglobin, NYHA class, diabetes, ejection fraction, echo-derived pulmonary artery systolic pressure and Doppler derived cardiac output. Variables from the univariate analysis found to be significant at *p* ≤ 0.1 were combined in the multiple variable analysis. The study population was divided into tertiles based on LVOT VTI and survival was compared between groups using Kaplan Meier and Cox regression analysis. Study data was analyzed using IBM SPSS software (IBM SPSS, version 21.0, IBM, Rochester, Minnesota).

## Results

### Study sample

Twenty-six thousand one hundred thirty-five TTE studies were performed during the selected time period; of these, 265 studies were identified with LVOT VTI < 10 cm; duplicate studies from the same subject (*n* = 125), subjects without at least a year of follow up (*n* = 22), heart rate > 120 beats per minute (*n* = 7), acute myocardial infarction at time of TTE (*n* = 6), pulmonary arterial hypertension (*n* = 1) and alternative causes for shock (*n* = 4) were excluded. The study sample consisted of 100 subjects; all subjects carried a diagnosis of heart failure (age 73.5 ± 14.7 years, 72% male, mean ejection fraction 28.9%). More than 60% of the study population had ischemic cardiomyopathy as the cause of their heart failure, 18% had dilated, non-ischemic cardiomyopathy, and the remaining 20% had tachycardia-induced cardiomyopathy (10%), viral myocarditis (5%), drugs/toxins (4%) and postpartum cardiomyopathy (1%). Other baseline subject characteristics are described in Table 1. A total of thirty events occurred (28 deaths and 2 LVADs) over 1 year of follow up from TTE study. No cardiac transplants were identified. When divided into LVOT VTI tertiles, ejection fraction demonstrated statistical significant across tertiles, (*p* = 0.024).

**Table 1:** Baseline characteristics

	Total study population (*n* = 100)	LVOT VTI < 8.1 cm, lowest tertile (*n* = 34)	LVOT VTI 8.1-9.0 cm, median tertile (*n* = 33)	LVOT VTI > 9.0 cm, highest tertile (*n* = 33)	*P*-value
Clinical characteristics					
Age (years)	73.53 ± 14.7	71.26 ± 15.3	74.82 ± 15.1	74.58 ± 13.8	0.547
Gender (% male)	72	79.4	75.8	60.6	0.194
Diabetes	27%	29.4%	24.2%	27.3%	0.892
Hypertension	57%	52.9%	51.5%	66.7%	0.388
Atrial Fibrillation	68%	77%	62%	67%	0.485
Prior revascularization	50%	56%	49%	45%	0.679
NYHA Class					0.643
I	1.1%	3.1%	0%	0%	
II	41.5%	40.6%	33.3%	51.7%	
III	45.7%	43.8%	54.5%	37.9%	
IV	11.7%	12.5%	12.1%	10.3%	
Smoking	14%	11.8%	10%	21.2%	0.328
Alcohol	17%	15%	24%	12%	0.385
Illicit drugs	4%	6%	3%	3%	0.797
Medical Therapy					
Diuretics	68.7%	72.7%	72.7%	60.6%	0.472
ACE/ARB	57.6%	64.7%	51.5%	56.3%	0.541
Beta blocker	84%	82.4%	84.8%	84.8%	0.949
Digoxin	28.3%	27.3%	30.3%	27.3%	0.951
Spironolactone	32%	35.3%	39.4%	21.2%	0.251
Statin	45.4%	41.2%	51.6%	43.8%	0.683
CRT	31.6%	31.3%	43.3%	21.2%	0.169
ICD	42.9%	51.5%	50.0%	27.3%	0.084
Objective Parameters					
SBP (mmHg)	114.57 ± 16.8	111 ± 17	113 ± 16	119 ± 17	0.128
DBP (mmHg)	72.00 ± 14.4	70 ± 16	71 ± 14	74 ± 13	0.581
HR (beats per minute)	82.93 ± 16.3	85.6 ± 18	80.9 ± 15	82.1 ± 16	0.481
Weight (kg)	76.6 ± 19.2	77.2 ± 16	80 ± 24	73.1 ± 17	0.391
Body mass index	25.8 ± 5	25.8 ± 5	26.4 ± 6	25.2 ± 5	0.657
Hgb (g/dL)	13.28 ± 2.0	13.7 ± 1.7	12.9 ± 1.7	13.2 ± 2.5	0.205
Creatinine (mg/dL)	1.40 ± 0.6	1.51 ± 0.8	1.46 ± 0.6	1.21 ± 0.4	0.101
Blood urea nitrogen (mg/dL)	27.0 ± 14	25.8 ± 10	30.5 ± 18	24.9 ± 13	0.221
Glomerular filtration rate < 60 ml/min	60.2%	67.7%	51.5%	62.1%	0.403
Sodium (mEq/L)	137 ± 4	137 ± 4	137 ± 3	138 ± 3	0.270
Cholesterol, total (mg/dL)	149 ± 41	142 ± 35	147 ± 41	158 ± 46	0.286
QRS > 120 msec	51.2%	61.3%	46.2%	44.4%	0.364
Ejection fraction (%)	28.9 ± 17	25.1 ± 17	26.4 ± 11	35 ± 19	0.024
Mean PA pressure (mmHg)	47.7 ± 15	47.8 ± 16	48.2 ± 13	47.1 ± 15	0.961
LV end diastolic dimension (cm)	5.81 ± 1.3	6.06 ± 1.2	5.94 ± 1.1	5.42 ± 1.5	0.092
Aortic Stenosis	11.1%	15.2%	10.0%	10.0%	0.900
Aortic Insufficiency	16.2%	24.2%	21.2%	3.0%	0.060
Tricuspid Regurgitation*	51.5%	54.5%	51.5%	48.4%	0.738
Mitral Regurgitation*	61%	55.9%	75.8%	51.5%	0.302

Legend: *SD* standard deviation, *NYHA* New York Heart Association; Diuretics furosemide, toresmide, bumetanide, *ACE* angiotensin converting enzyme, *ARB* angiotensin receptor blocker, *CRT* cardiac resynchronization therapy, *ICD* implantable cardiac defibrillator, *SBP* systolic blood pressure, *DBP* diastolic blood pressure, *bpm* beats per minute, *GFR* glomerular filtration rate, *PA* pulmonary artery, *LV* left ventricle

*denotes meaningful valve disease classified as greater than mild

### Outcome group analysis

Comparison of subjects meeting primary endpoints (death or LVAD or transplant, *n* = 30) versus event-free subjects (*n* = 70) identified the following variables as significantly associated with death and LVAD placement: LVOT VTI (*p* = 0.039), older age (*p* = 0.001), higher NYHA class (*p* = 0.014), higher pulmonary artery systolic pressure (53.0 ± 16.9 vs. 45.3 ± 12.6 mmHg; *p* = 0.019), higher blood urea nitrogen (33.8 ± 15.4 vs 24.1 ± 12.5, *p* = 0.001), lower hemoglobin (12.7 ± 1.4 vs. 13.5 ± 2.2 g/dL; *p* = 0.030), and glomerular filtration rate < 60 ml/min (75.9% vs 53.1%, *p* = 0.038), results summarized in Table 2.

**Table 2:** Comparison of baseline variables by outcome group: Primary outcome vs Event free subgroups

Variable	Primary outcome group (*n* = 30)	Event-free group (*n* = 70)	*p*-value
LVOT VTI	8.0	8.5	0.039
Ejection fraction (%)	32.3	27.4	0.169
Doppler derived cardiac output (L/min)	2.29	2.44	0.217
Age, years	80.1 ± 11.3	70.7 ± 15.1	0.003
NYHA class (% in stage III or IV)	80%	48.60%	0.014
Hemoglobin, g/dL	12.7 ± 1.4	13.5 ± 2.2	0.030
Blood urea nitrogen, g/dL	33.8 ± 15.4	24.1 ± 12.5	0.001
GFR <60 ml/min	75.90%	53.10%	0.038
Pulmonary artery pressure, mmHg	53.0 ± 16.9	45.3 ± 12.6	0.019

Primary outcome was met in 30 subjects (28 death and 2 LVADs); Event free subjects were those alive at one year of follow up from TTE and without requirement for mechanical circulatory support

Abbrev: *GFR* glomerular filtration rate, *LVOT VTI* Left ventricular outflow tract velocity time integral, *NYHA* New York Heart Association

### Univariate outcomes prediction analysis

A set of predefined variables including age, gender, presence of diabetes, NYHA class, hemoglobin, blood urea nitrogen, glomerular filtration rate < 60 ml/min, pulmonary artery systolic pressure, ejection fraction, LVOT VTI, and Doppler derived cardiac output were assessed (Table 3). Significant variables that were associated with death and LVAD implatation included LVOT VTI, hazard ratio (HR) of 0.729 (95% confidence interval [CI] 0.55 - 0.96, *p* = 0.024), age (HR 1.05, CI 1.02 - 1.09, *p* = 0.001), NYHA class (HR 2.13, CI 1.26 - 3.60, *p* = 0.005), blood urea nitrogen (HR 1.03, CI1.01 - 1.05, *p* = 0.005), and glomerular filtration rate (HR 2.437, CI 1.04 - 5.71, *p* = 0.029).

**Table 3:** Univariate and multiple variable analyses of factors related to death or LVAD placement

	Hazard ratio (95% CI)	*P*-value
Univariate analysis		
LVOT VTI, cm	0.729 (0.55 - 0.96)	0.024
Age, years	1.05 (1.02 - 1.09)	0.001
Male gender	1.66 (0.678 - 4.058)	0.245
Diabetes	1.38 (0.65 - 2.95)	0.416
NYHA class	2.13 (1.26 - 3.60)	0.005
Hemoglobin, g/dL	0.876 (0.74 - 1.04)	0.107
Blood urea nitrogen, g/dL	1.03 (1.01 - 1.05)	0.005
Glomerular filtration rate < 60 ml/min	2.437 (1.04 - 5.71)	0.029
Pulmonary artery pressure, mmHg	1.03 (1.01 - 1.06)	0.010
Ejection fraction	1.01 (0.99 - 1.03)	0.179
Doppler derived cardiac output, L/min	0.677 (0.378-1.213)	0.190
Multivariate analysis		
Model 1: LVOT VTI + age	0.661 (0.502 – 0.871)	0.003
Model 2: Model 1 + Pulmonary artery pressure and Ejection fraction	0.619 (0.452 – 0.849)	0.003
Model 3: Model 2 + Blood urea nitrogen, glomerular filtration rate < 60 ml/min and NYHA class	0.590 (0.415 – 0.838)	0.003

Legend: *HR* hazard ratio, *CI* confidence interval, *LVAD* left ventricular assist device, *LVOT VTI* left ventricular outflow tract velocity time integral; other abbreviations described in Table 1

### Multivariate analysis

In the multivariate model, all variables in the univariate analysis with *p* value ≤0.1 and the prespecified addition of ejection fraction were included (Table 3). In model 1 LVOT VTI was adjusted by age; model 2 consisted of all variables in model 1 with the addition of echocardiographic factors including ejection fraction and echo derived pulmonary artery systolic pressure. Model 3 consisted of all variables in model 2 with the addition of NYHA class, glomerular filtration rate < 60 ml/min and blood urea nitrogen. In all models, lower LVOT VTI remained significantly associated with death and LVAD implantation (Table 3).

### Survival analysis

**Fig. 2:**
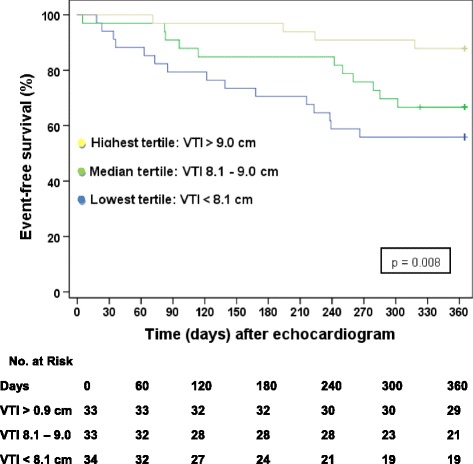
Kaplan Meier Survival Analysis by LVOT VTI Tertile

Subjects were divided into three groups based on LVOT VTI tertile: lowest tertile (LVOT VTI < 8.1 cm, *n* = 34), median tertile (LVOT VTI 8.1-9.0 cm, *n* = 33), and upper tertile (LVOT VTI > 9.0 cm, *n* = 33). Event free survival rates were 55.9% for the lowest LVOT VTI tertile, 66.7% for the median tertile, and 87.9% for the upper tertile, *p* = 0.008 (Fig. 2. Kaplan Meier Survival analysis by LVOT VTI Tertile). The hazard ratio for death and LVAD placement for the lowest tertile (LVOT VTI > 8.1 cm, *n* = 34) in comparison to the upper two tertiles (LVOT VTI > 8.1 cm, *n* = 66) was 4.755 (CI 1.576-14.348, *p* = 0.006), and the HR was 12.680 (CI 2.638 – 60.949, *p* = 0.002) when adjusted for age, creatinine, ejection fraction, pulmonary artery systolic pressure and NYHA class (Table 4).

**Table 4:** Cox proportional hazard analysis by LVOT VTI tertile

	Lowest tertile (LVOT VTI < 8.1, *n* = 34) vs Upper 2/3 s study population (*n* = 66)	*P*-value
Unadjusted analysis	4.755 (CI 1.576 - 14.348)	0.006
Adjusted analysis*	12.680 (CI 2.638 – 60.949)	0.002

*Adjusted for Age, Creatinine, Ejection fraction, Pulmonary artery systolic pressure and NYHA class

## Discussion

Because extremely low LVOT VTI was the focus of our investigation, a cut off of <10 cm was used for subject inclusion. Similar to previously described heart failure cohorts, our study population was predominantly male (72 out of 100), elderly (mean age, 73.5 years) with systolic CHF due to ischemic cardiomyopathy (mean EF, 28%). Our findings demonstrate that extremely diminished LVOT VTI was robustly associated with the combination of 12-month death and LVAD implantation. In the multivariate analysis, LVOT VTI was most predictive of adverse outcomes [HR 0.589 (95% CI 0.41 - 0.83), *p* = 0.003], hazard ratio of less than one indicating that higher LVOT VTI correlates with better outcomes, other significant variables being older age [HR 1.04 (95% CI 1.004 - 1.095, *p* = 0.033] and higher echo-derived systolic pulmonary artery pressure [HR 1.03 (95% CI 1.005 - 1.065, *p* = 0.022].

When comparing cohort tertiles, the unadjusted and adjusted hazard ratios for LVOT VTI were even more predictive, with an unadjusted mortality and LVAD likelihood ratio of 4.755 (95% CI 1.576 - 14.348) in the lowest LVOT VTI tertile compared with the rest of the study group and an adjusted likelihood ratio of 12.680 (95% CI 2.638 – 60.949).

Several reasons may explain why low LVOT VTI correlates closely with adverse clinical outcomes. LVOT VTI provides enhanced prognostic information over ejection fraction, as it focuses on forward cardiac output which at times maybe normal even in compensated heart failure patients with low ejection fraction. Low cardiac output is a known precursor to overt cardiogenic shock, multi-organ dysfunction and death [18]. Whereas the accuracy of Doppler derived cardiac output is primarily limited by errors in determining the cross sectional area of the LVOT, as defined by the formula *πr* ^2^, utilizing LVOT VTI alone rather than Doppler derived cardiac output eliminates this source of error. In patients who are tachycardic due to cardiogenic shock and poor LV function, rapid heart rate partially offsets the decline LV function, allowing for maintenance of cardiac output in the setting of a sick ventricle, however LVOT VTI remains depressed. Thus, in cases of low cardiac output with compensatory tachycardia, LVOT VTI may be a very sensitive predictor of cardiogenic shock and impaired ability to meet systemic tissue perfusion and metabolic demands. Because LVOT VTI is an easily obtainable and reproducible measurement, we propose that LVOT VTI may be a useful and accessible tool to identify heart failure patients with very low cardiac output and who may benefit from advanced heart failure therapies appropriate for end-stage HF.

## Conclusions

As numerous therapies emerge that increase cardiac output for patients with advanced heart failure [19], there is an ever greater need to identify those who stand to benefit most from such therapies [4]. LVOT VTI is an easily available non-invasive tool that identifies patients at highest risk for decreased survival at one year, thus allowing for earlier identification and advanced treatment.

### Study limitations and future directions

Accurate determination of LVOT VTI assumes laminar flow and is affected by LVOT abnormalities such as severe aortic regurgitation, hypertrophic obstructive cardiomyopathy, systolic anterior motion of the anterior mitral leaflet, and subaortic stenosis; subjects with these diagnoses were excluded in our study as LVOT VTI is not an accurate predictor of forward cardiac flow in these settings [5].

Our study was designed as a novel proof-of-concept study, no prior study to our knowledge at the time of this writing has examined the relationship between extremely low LVOT VTI and outcomes in advanced heart failure. Given the retrospective nature of this study, findings should be confirmed a larger, prospective cohort.

## Abbreviations

CI: 95% confidence interval
CO: Doppler derived cardiac output
EF: Ejection fraction
EMR: Electronic medical record
GFR: Glomerular filtration rate
HR: Hazard ratio
HR: Heart rate
LVAD: Left ventricular assist device
LVOT VTI: Left ventricular outflow tract velocity time integral
NYHA class: New York Heart Association class
PASP: Pulmonary artery systolic pressure,
SV: Stroke volume
TTE: Transthoracic echocardiogram
